# Preserving Continuity of Cancer Therapy in Patients with Hematologic Malignancies and Chronic Subdural Hematomas: Comparative Outcomes of Middle Meningeal Artery Embolization and Surgery

**DOI:** 10.3390/cancers18172824

**Published:** 2026-09-01

**Authors:** Esteban Ramirez-Ferrer, Juan P. Zuluaga-Garcia, Maria A. Sierra, Danielle Hammond, Hagop M. Kantarjian, Jeffrey S. Weinberg, Chibawanye I. Ene, Shaan M. Raza, Frederick F. Lang, Peter T. Kan, Stephen R. Chen, Christopher C. Young

**Affiliations:** 1 Department of Neurosurgery, The University of Texas MD Anderson Cancer Center, Houston, TX 77030, USA; eramirez14@mdanderson.org (E.R.-F.); jpzuluaga@mdanderson.org (J.P.Z.-G.); jweinberg@mdanderson.org (J.S.W.); cene@mdanderson.org (C.I.E.); smraza@mdanderson.org (S.M.R.); flang@mdanderson.org (F.F.L.); 2 School of Medicine, Universidad del Rosario, Bogotá 110221, Colombia; mariaaleja.sierra@urosario.edu.co; 3 Department of Leukemia, The University of Texas MD Anderson Cancer Center, Houston, TX 77030, USA; dhammond@mdanderson.org (D.H.); hkantarjian@mdanderson.org (H.M.K.); 4 Department of Neurosurgery, University of Texas Medical Branch, Galveston, TX 77555, USA; ptkan@utmb.edu; 5 Department of Interventional Radiology, The University of Texas MD Anderson Cancer Center, Houston, TX 77030, USA; srchen@mdanderson.org

**Keywords:** chronic subdural hematoma, middle meningeal artery embolization, leukemia, lymphoma, multiple myeloma, recurrence, burr hole, craniotomy

## Abstract

**Background/Objective**: Hematologic cancer patients are at high risk of developing chronic subdural hematomas (cSDH), and traditional treatment with surgery is potentially risky in this patient population, with associated coagulopathy and thrombocytopenia. Neurointerventional endovascular middle meningeal artery embolization (MMAE) is emerging as a novel minimally invasive treatment method for cSDH, and its efficacy and complication profile has not been evaluated in patients with hematologic cancers. **Methods**: We conducted a retrospective cohort study of adults with hematologic malignancies and cSDH between 2015 and 2025 and compared the efficacy and safety of treatment with MMAE versus open surgery. Variables were abstracted from the medical records. Primary outcomes were necessity for rescue treatment within 180 days after the index procedure and time to restart systemic cancer therapy among patients whose treatment was held due to subdural hematoma. Inverse probability of treatment weighting (IPTW) was applied using covariate-balancing propensity scores to address confounding by indication. Time-to-event outcomes were analyzed using weighted Cox proportional hazards models and Kaplan–Meier methods. **Results**: A total of 67 patients with hematologic malignancies and cSDH were included (37 treated with MMAE and 30 with surgical evacuation). After IPTW, baseline covariates were well balanced between groups. MMAE was associated with a significantly lower risk of requiring rescue treatment than surgery (HR, 0.19; 95% CI, 0.05–0.81). This association persisted after accounting for the competing risk of death (Fine–Gray subdistribution HR, 0.21; 95% CI, 0.06–0.77). Prolonged Activated Partial Thromboplastin Time was associated with a higher risk of requiring rescue treatment. The median time to resume systemic therapy was significantly shorter following MMAE (13.5 days; 95% CI, 9–26) than after surgery (27.0 days; 95% CI, 22–50) (*p* = 0.005). **Conclusions**: In patients with hematologic malignancies and symptomatic cSDH, MMAE was associated with fewer rescue interventions and earlier resumption of systemic therapy compared with surgical evacuation, suggesting that embolization may represent an effective and minimally invasive treatment strategy that preserves continuity of oncologic care in this high-risk population.

## Simple Summary

Patients with blood cancers are at high risk of developing chronic subdural hematomas (cSDH). These blood clots between the brain and skull exert pressure on the underlying brain, resulting in neurologic symptoms and often interrupting their cancer treatment. This study compares two treatment approaches: a minimally invasive procedure that blocks the blood vessels feeding the chronic membrane and causing bleeding versus traditional open surgery to drain it. The researchers find that the minimally invasive approach resulted in fewer complications and allowed patients to resume oncologic treatment approximately two weeks earlier than surgery. This is clinically important because any delay in cancer treatment can negatively affect patient outcomes. These findings suggest that the minimally invasive approach may be a reasonable option for cancer patients with cSDH, as it minimizes interruption of cancer therapy while effectively treating the cSDH.

## 1. Introduction

Patients with hematologic malignancies (i.e., leukemia, lymphomas, or myelomas) are particularly prone to developing chronic subdural hematomas (cSDH) due to higher rates of thrombocytopenia, platelet dysfunction, anticoagulant use, and treatment-related coagulopathy, which increases the bleeding risk while simultaneously limiting treatment options [1,2]. In this setting, standard surgical evacuation—although effective for mass effect—is complicated by high perioperative transfusion requirements (with platelet thresholds of >100 × 10^3^/μL recommended for neurosurgical procedures), substantial recurrence rates (30-day reoperation rates of 35% in this population), and postoperative morbidity that can delay timely delivery of systemic cancer therapy [1,3]. Maintaining oncologic therapies within narrow therapeutic windows is not simply a logistical goal but a prognostic determinant in many hematologic malignancies, with worse 1-year progression-free survival (PFS) and overall survival (OS) rates in those where therapy has been interrupted, underscoring the clinical consequences of interruption [4].

**Figure 1:**
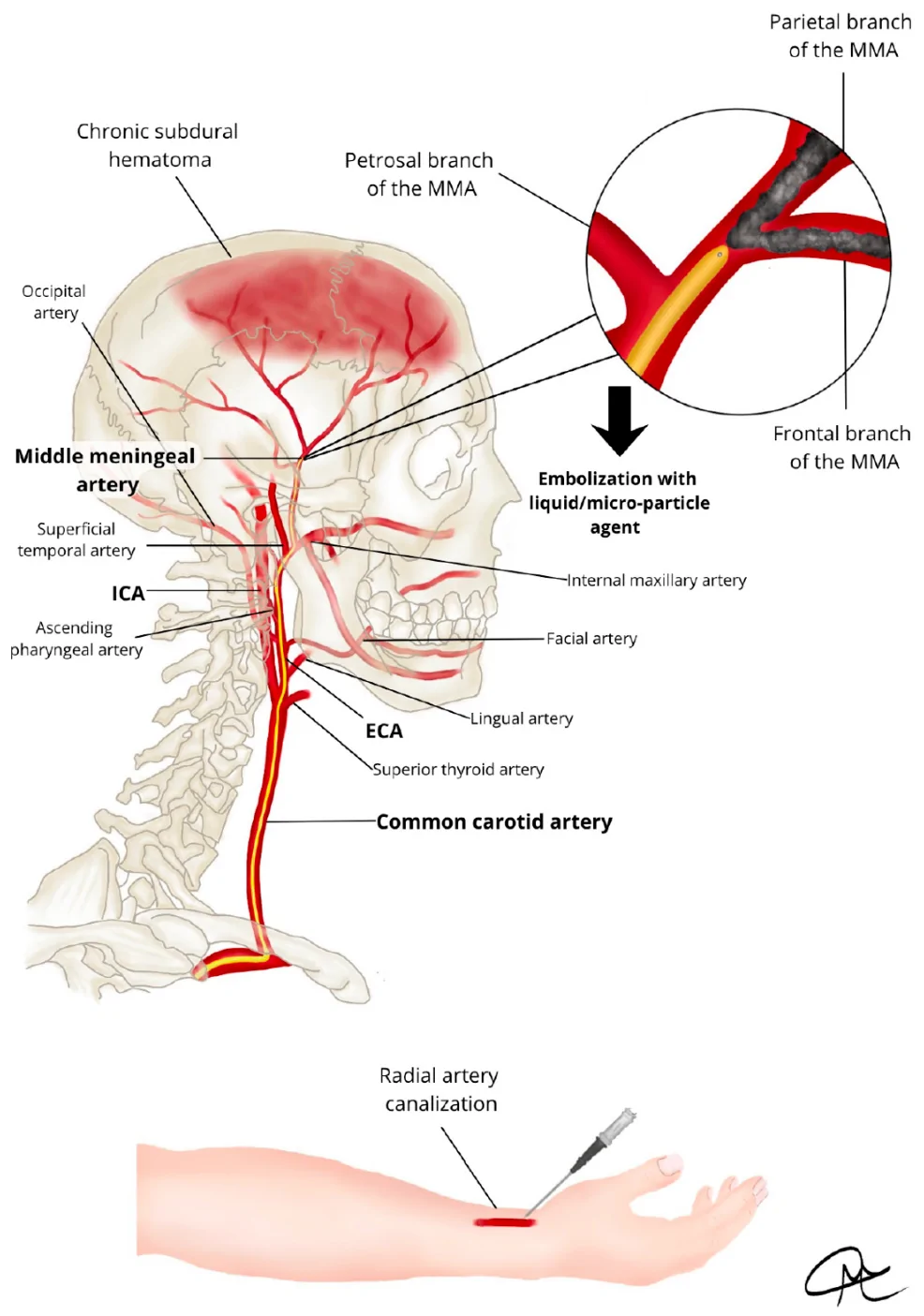
Middle meningeal artery embolization diagram.

Neurointerventional endovascular middle meningeal artery embolization (MMAE) has emerged as a mechanism-directed strategy for cSDH, targeting the vascularized neo-membranes that sustain hematoma persistence and recurrence [5,6]. It is a minimally invasive endovascular procedure performed in a biplane angiography suite. Through a standard transradial or transfemoral arterial approach, a microcatheter is navigated into the middle meningeal artery (MMA), a branch of the maxillary artery that enters the skull through the foramen spinosum and arborizes across the inner surface of the dura mater [7]. Once positioned distally within the MMA, embolic material (typically liquid embolic agents or polyvinyl alcohol particles) is deployed to occlude the vessel and its branches, which targets the underlying pathophysiology of cSDH: rather than simply evacuating accumulated blood, MMAE addresses the vascularized neo-membranes that form within the subdural space in response to the initial hemorrhage. These membranes, derived from dural border cells and richly supplied by MMA branches, contain highly permeable, fragile sinusoids that sustain a cycle of microhemorrhage, osmotic fluid accumulation, and hematoma expansion [8]. By devascularizing these neo-membranes, MMAE interrupts this self-perpetuating cycle, allowing the hematoma to be gradually reabsorbed (Figure 1).

In non-oncologic SDH populations, randomized controlled trials have demonstrated that adjunctive MMAE significantly reduces treatment failure compared with standard surgical management alone, with a 64–72% reduction in the relative risk of reoperation [5,6,9,10,11]. MMAE is also emerging as a potential standalone treatment for symptomatic cSDH and is currently being evaluated in clinical trials (NCT06347796) [12]. In oncology cohorts, early experience suggests that MMAE is technically feasible, has a low rate of adverse events, and may reduce the need for subsequent intervention, even among patients with hematologic malignancies or peri-treatment coagulopathy [3]. Importantly, our institutional data from broader cancer populations indicate that MMAE is associated not only with fewer recurrences but also with earlier resumption of oncologic therapy compared with surgical drainage—an outcome particularly relevant when delays threaten disease control [13].

Despite these encouraging findings, comparative effectiveness data specifically focused on hematologic malignancies—where coagulopathy is common and uninterrupted systemic therapy is paramount—remain limited. This study evaluates our experience with MMAE versus surgical evacuation for cSDH in patients with hematologic malignancies, emphasizing (1) the need for rescue treatment/reintervention and (2) time to restart systemic cancer therapy as dual, clinically meaningful endpoints.

## 2. Materials and Methods

### 2.1. Study Design and Patient Selection

**Figure 2:**
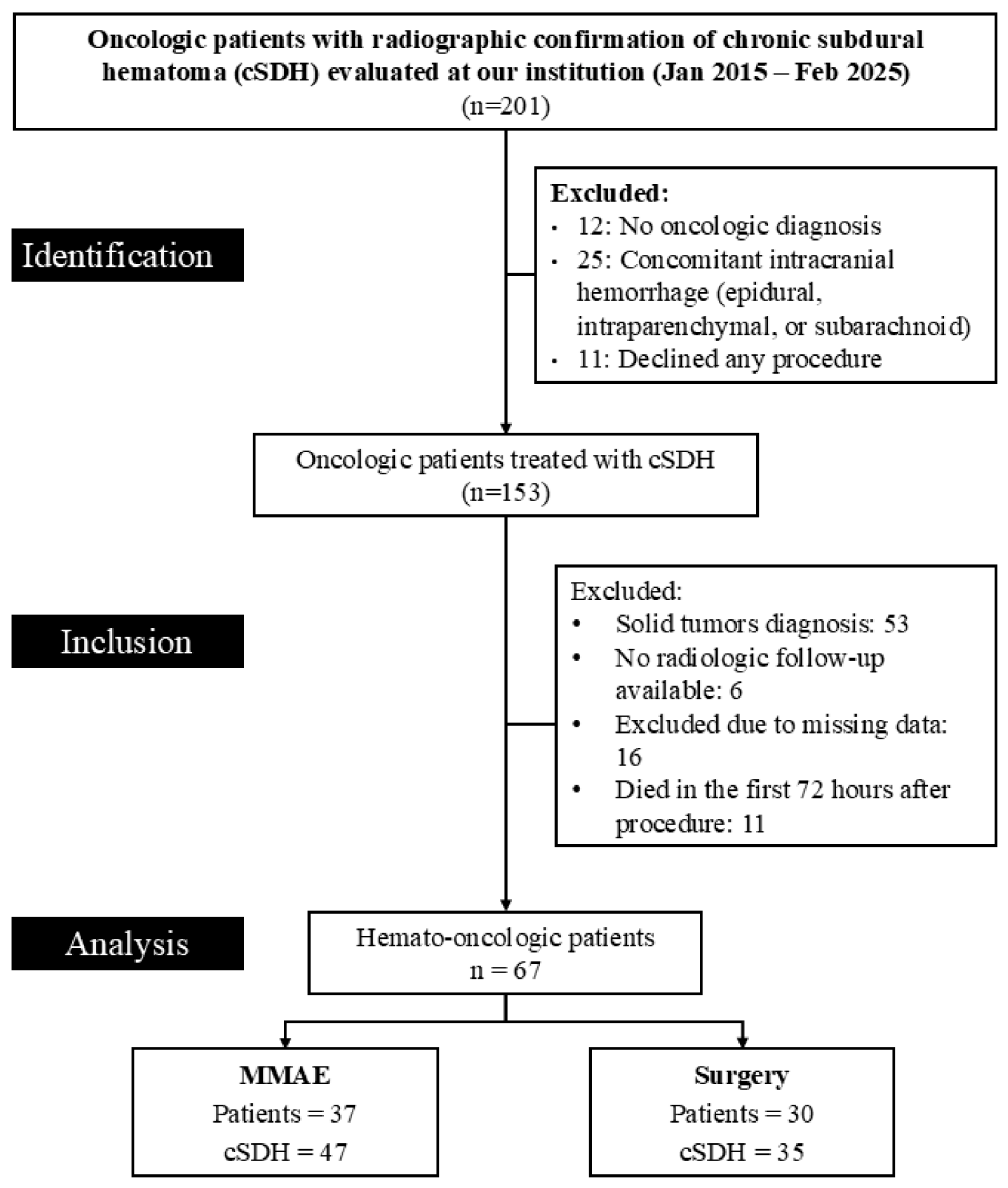
STROBE diagram.

A focused retrospective cohort study of adults with hematologic malignancies and radiographically confirmed cSDH, treated at a single tertiary cancer center between January 2015 and February 2025, was conducted. The study was approved by the institutional review board with a waiver of informed consent. Patients were identified from institutional neurosurgical and interventional radiology databases. Inclusion criteria were a hematologic malignancy diagnosis, cSDH (i.e., chronic and subacute) on CT/MRI, and availability of pre- and postoperative clinical and imaging data. Exclusion criteria were remission of hematologic malignancy, concomitant intracranial hemorrhage (epidural, intraparenchymal, or subarachnoid), refusal of any procedure, no radiologic follow-up, missing clinical data, indication for emergent surgical evacuation, or death within 72 h of the index procedure. The selection process is summarized in a STROBE-style flow diagram (Figure 2).

### 2.2. Data Collection

Demographic, clinical, imaging, and treatment variables were collected from the electronic medical records. Performance status was documented using Karnofsky Performance Status (KPS). Hematologic diagnoses were grouped into myeloid neoplasms, B-cell lymphomas, lymphoid leukemias, plasma cell dyscrasias, and T/NK cell lymphomas. Laboratory variables included preoperative platelet count (≤50, 50–100, >100 × 10^9^/L), International Normalized Ratio (INR), Activated Partial Thromboplastin Time (aPTT), Prothrombine Time (PT), and hematocrit. Antithrombotic exposure included antiplatelet and/or anticoagulant therapy at presentation. Transfusion therapy was quantified as the number of units administered within 48 h before and after the procedure; one platelet apheresis was standardized to six pooled units.

Imaging measurements were obtained at prespecified timepoints, namely, preoperative, and early postoperative (within the first 48 h after the procedure), and at 1 month, 3 months, and 6 months, by two blinded reviewers. Maximum cSDH diameter was measured on coronal images when available; midline shift was measured on axial images as the perpendicular deviation of the septum pellucidum from a line connecting the anterior and posterior falx. Bilateral values were recorded independently. Subdural membranes, acute components, and herniation signs were recorded when present. Systemic oncologic therapy was considered *held* when paused due to SDH and *restarted* on the documented resumption date. For daily regimens, the hold date was the SDH diagnosis date; for cyclic regimens, it was the planned start date of the next cycle.

### 2.3. Primary Outcome

The primary clinical outcome was the need for rescue treatment (i.e., repeat embolization or surgical reintervention for persistent or recurrent cSDH) within 180 days of the index procedure. Rescue was triggered by combined clinical and radiographic deterioration; radiographic progression alone prompted closer surveillance rather than reintervention. The principal oncologic outcome was time to restart systemic therapy among patients whose cancer treatment had been held for cSDH.

### 2.4. Statistical Analysis

Continuous variables are reported as median (IQR) and compared using the Wilcoxon rank-sum test; categorical variables are reported as *n* (%) and compared using Pearson’s χ^2^ or Fisher’s exact test. To address confounding by indication, covariate-balancing propensity scores for MMAE versus surgery were used to construct inverse probability of treatment weights (IPTW) for the average treatment effect [14]. The propensity model included age ≥ 65 years, KPS > 70, platelet category, INR > 1.5, active systemic therapy, any antithrombotic therapy, midline shift ≥ 5 mm, and acute/mixed cSDH. Weights were truncated at the 5th and 95th percentiles, and balance was assessed using standardized mean differences (target < 0.10). We prioritized midline shift as the imaging covariate in the propensity model because it is a clinically salient measure of mass effect that strongly influences treatment selection and is correlated with hematoma burden; including both midline shift and thickness risked collinearity given the sample size.

For time-to-rescue, Cox proportional hazards models with robust (sandwich) standard errors were fitted using IPTW; covariates included procedure, age ≥ 65 years, KPS > 70, midline shift ≥ 5 mm, INR > 1.5, aPTT > 35 s, and platelet category (reference > 100 × 10^9^/L). Proportional hazards were assessed visually and with Schoenfeld residuals. Time to systemic therapy restart was analyzed using Kaplan–Meier curves and log-rank tests; medians and 95% confidence intervals were reported, and a weighted Cox model by procedure was used in a sensitivity analysis. Given the 13 rescue events, the multivariable model is reported as exploratory, and a parsimonious procedure-only model served as the primary comparison. Cumulative incidence of rescue was estimated with the Aalen–Johansen method treating death as a competing risk, and groups were compared with a Fine–Gray subdistribution hazard model [15]. The 30-, 90-, and 180-day mortality and overall survival were summarized by group. Eleven patients who died within 72 h of the index procedure had solid tumors and fell outside the hematologic malignancy cohort; in all cases, death was driven by multiple comorbidities rather than the procedure or the hematoma, and these patients were therefore not included. SDH thickness and midline shift over time were displayed using box-and-jitter plots by procedure. All analyses were performed in R 4.4.3 [16].

## 3. Results

### 3.1. Study Population

A total of 67 patients with hematologic malignancies and non-acute SDH were included, of whom 37 underwent MMAE and 30 underwent surgical hematoma evacuation (Figure 2). Baseline demographic characteristics, including age, sex, body mass index, Glasgow Coma Scale (GCS), and preoperative KPS, were comparable between groups. Hematologic diagnoses included myeloid neoplasms, lymphoid leukemias, lymphomas, and plasma cell dyscrasias, with no significant differences in diagnostic distribution (Table 1). Further clinical details regarding diagnoses and treatment data are provided in Supplementary Table S1.

Patients selected for surgical evacuation had greater radiographic severity, including larger median SDH diameter (median 17.6 vs. 13.2 mm; *p* = 0.005) and a larger median midline shift (median 8.9 vs. 0.0 mm; *p* < 0.001). Coagulopathy patterns also differed: thrombocytopenia and mixed coagulopathies were more prevalent in the MMAE group (*p* < 0.001), whereas preprocedural correction of coagulopathy was more frequent among surgically treated patients (*p* < 0.001). Procedure duration was significantly shorter for MMAE (median 60 vs. 135 min; *p* < 0.001). New postoperative neurologic deficits (8.1% vs. 30.0%), wound complications (0% vs. 10.0%), and hospital length of stay (median 4 vs. 19 days) were lower after MMAE (Table 1).

After application of IPTW, baseline covariates prespecified in the propensity model—including age, performance status, platelet count, antiplatelet/anticoagulation therapy use (i.e., antithrombotic therapy), radiographic severity (midline shift and maximal diameter), and presence of mixed SDH components—were balanced between treatment groups, with standardized mean differences below prespecified thresholds (Table 2, Supplementary Figure S1).

### 3.2. Rescue Treatment

Rescue treatment within 180 days occurred less frequently after MMAE than after surgical evacuation (8.1% vs. 33.3%, *p* = 0.013) (Table 1). In IPTW-weighted exploratory Cox proportional hazards models, MMAE was associated with a lower hazard of rescue treatment compared with surgery after multivariate analysis (HR, 0.19; 95% CI, 0.05–0.81; *p* = 0.024) (Table 3). On the contrary, prolonged aPTT (>35 s) was associated with a higher hazard of rescue treatment (HR, 5.38; 95% CI, 1.07–12.19; *p* = 0.041). Interestingly, platelet count ≤ 50 × 10^9^/L was associated with a lower hazard of rescue compared with platelet count > 100 × 10^9^/L (HR, 0.16; 95% CI, 0.03–0.87; *p* = 0.034).

**Figure 3:**
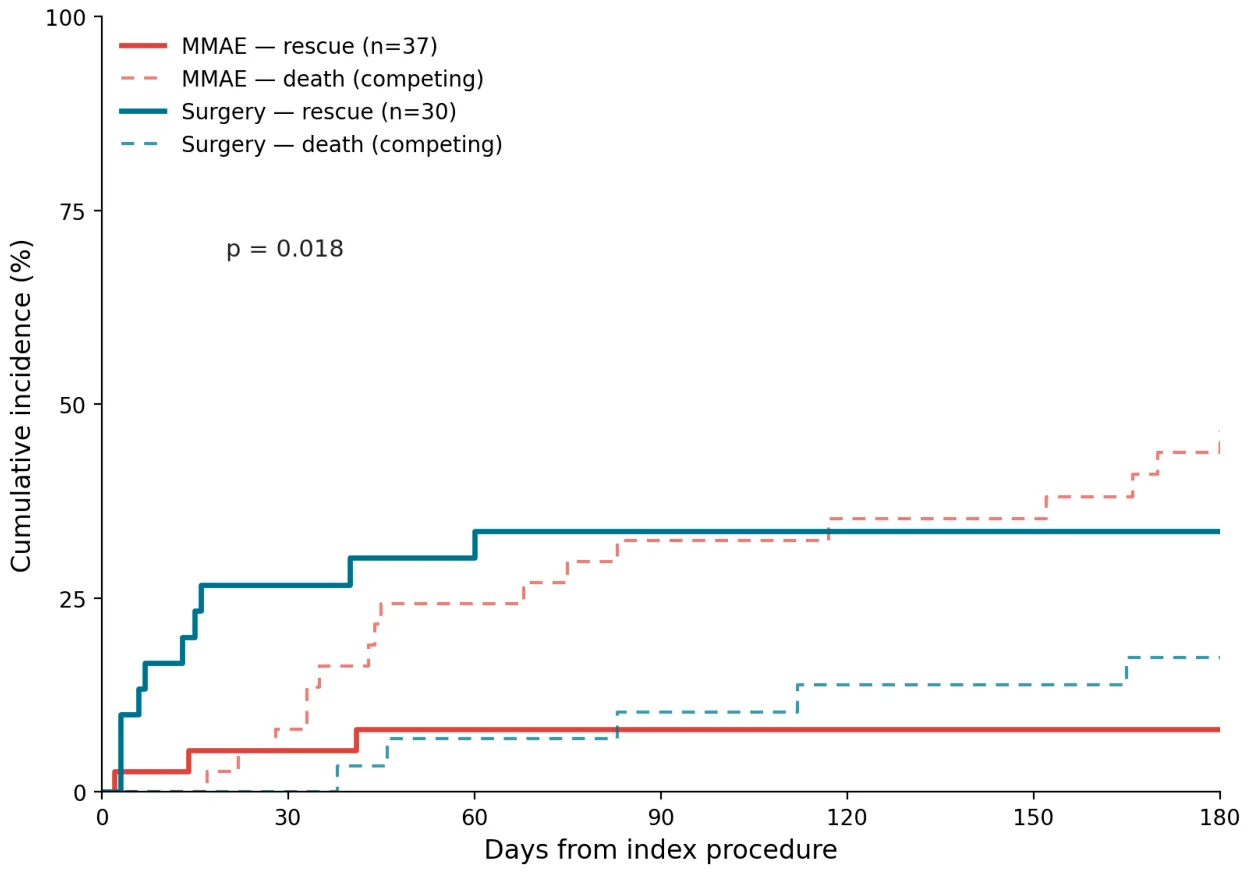
Cumulative incidence of rescue treatment with death as a competing risk.

Accounting for the competing risk of death, the 180-day cumulative incidence of rescue was 8.1% after MMAE versus 33.7% after surgery (Fine–Gray subdistribution HR, 0.21; 95% CI, 0.06–0.77) (Figure 3). Mortality at 30, 90, and 180 days was 8.1%, 35.1%, and 48.6% after MMAE versus 0%, 10.0%, and 23.3% after surgery, and median overall survival was 5.9 versus 10.4 months.

### 3.3. Resumption of Systemic Therapy

**Figure 4:**
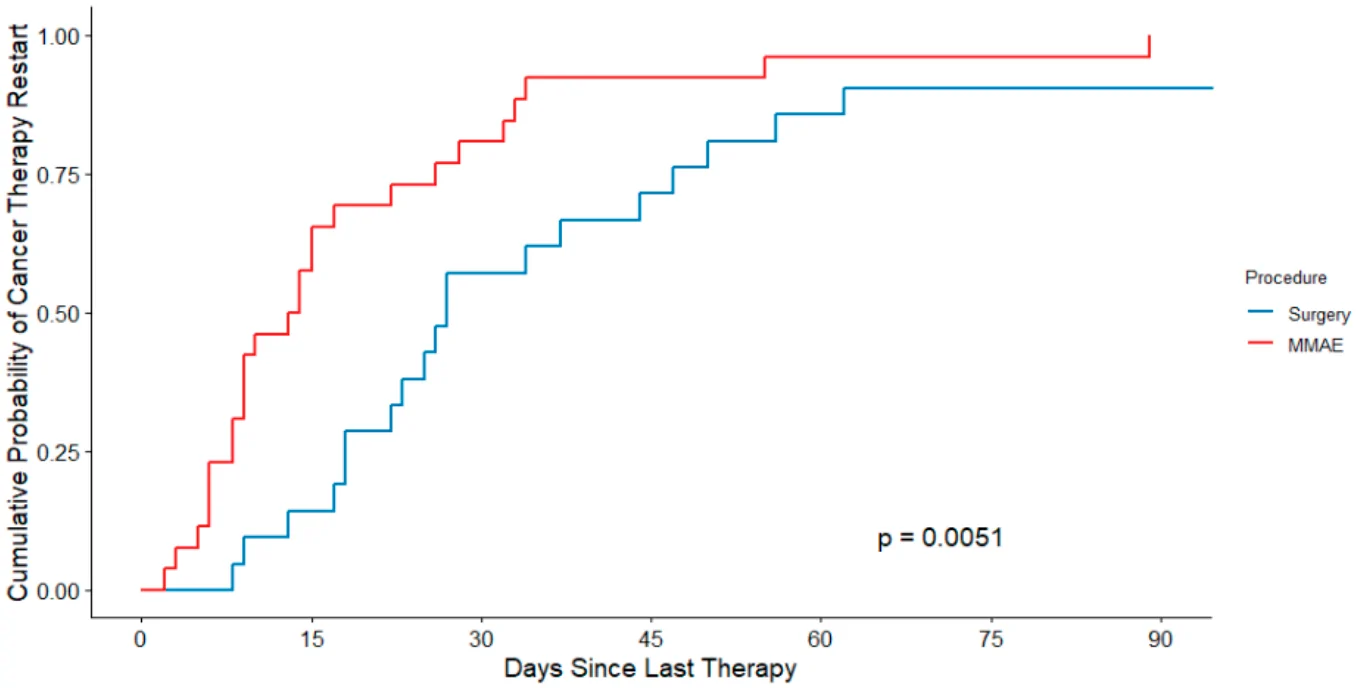
Time to cancer therapy resumption after SDH treatment: MMAE vs. surgery.

At presentation, the majority of patients in both groups were receiving active systemic cancer therapy—84% and 80% in the MMAE and surgery groups, respectively. Systemic therapy was held because of the cSDH in 26 of 37 MMAE and 22 of 30 surgical patients, and every patient in whom therapy was held ultimately resumed it. The time origin was the cSDH diagnosis date for daily regimens and the planned next-cycle date for cyclic regimens. Among these patients, the time to resume systemic therapy differed significantly by treatment strategy. Kaplan–Meier analysis demonstrated earlier and more frequent resumption of systemic therapy among patients treated with MMAE (13.5 days 95% CI, 9–26) compared to surgery (27 days 95% CI, 22–50) (*p* = 0.005) (Figure 4). This association persisted in sensitivity analyses using IPTW-weighted Cox proportional hazards models. Peri-procedural transfusion requirements were numerically lower following MMAE, although these differences did not reach statistical significance.

## 4. Discussion

In this retrospective cohort of patients with hematologic malignancies and non-acute cSDH, MMAE was effective compared to surgical evacuation. MMAE was associated with a significantly lower need for rescue treatment and a substantially shorter time to resumption of systemic cancer therapy compared with surgical evacuation. These findings underscore the potential role of MMAE as a management strategy that balances durable hematoma control with preservation of oncologic treatment continuity in a uniquely high-risk population.

Patients with hematologic malignancies represent a distinct subgroup within the broader non-acute cSDH population [17]. Treatment-related thrombocytopenia, qualitative platelet dysfunction, antiplatelet/anticoagulant use, and fluctuating coagulation profiles increase hemorrhagic risk, procedural complexity, and perioperative complications [1,18,19]. In the present cohort, patients treated with MMAE exhibited thrombocytopenia and mixed coagulopathies more frequently, whereas those undergoing surgical evacuation more often required preprocedural correction of coagulation abnormalities. Despite this adverse hematologic profile, MMAE was associated with fewer retreatments after adjustment for baseline differences, supporting its effectiveness even in the setting of impaired hemostasis. Interestingly, our results showed that severe thrombocytopenia (i.e., platelet count ≤ 50 × 10^9^/L) was associated with a lower risk of rescue treatment. However, this finding is best explained by collider bias from treatment selection rather than a biological effect. A baseline count ≤ 50 × 10^9^/L triggered near-universal peri-procedural platelet transfusion and correction (20 of 22 such patients were transfused), so the recorded baseline no longer reflected hemostasis at the time of the procedure, and post-transfusion counts are not routinely rechecked. Conditioning on the baseline category therefore conditions on a variable upstream of a corrective intervention, which can invert the apparent association.

The lower rate of rescue treatment observed after MMAE aligns with its proposed mechanism of action, which targets the vascularized membranes responsible for hematoma persistence and recurrence rather than focusing solely on clot evacuation and mechanical relief achieved by the surgical approach [9,20]. By addressing the underlying pathophysiology of non-acute subdural hematomas, MMAE may provide a more durable solution in patients prone to rebleeding, particularly in a coagulopathic context [3,6,9,21,22]. Notably, this association persisted in our study after IPTW, suggesting that differences in baseline radiographic severity or selection bias alone do not fully explain the observed effect, though the finding remains exploratory given the limited rescue events. Early mortality was higher after MMAE, consistent with its selection for the more systemically advanced patients; nonetheless, the lower incidence of rescue persisted after accounting for the competing risk of death (Fine–Gray subdistribution HR, 0.21), indicating that the finding is not an artifact of differential mortality.

Beyond neurosurgical outcomes, the oncologic implications of treatment strategy are particularly relevant in patients with hematologic malignancies. Interruptions or delays in systemic therapy have been associated with inferior disease control and survival in multiple hematologic cancers, particularly in aggressive leukemias and lymphomas, where treatment intensity and timing are critical [23,24,25]. In this study, patients undergoing MMAE resumed systemic therapy approximately two weeks earlier than those treated surgically. While causality cannot be inferred, this difference is clinically meaningful. It likely reflects the minimally invasive nature of embolization, shorter procedure times, reduced perioperative physiological stress, and avoidance of wound-related and hematologic considerations that may delay resumption of treatment after surgery.

This cohort is a focused hematologic malignancy subgroup analysis of our previously reported oncologic cohort; all 67 patients are contained within that 110-patient cohort (43 solid tumors and 67 hematologic malignancies), and the present analysis adds disease-specific coagulopathy, platelet, transfusion, and therapy-continuity data not examined previously [13]. In contrast to cancer-wide analyses, this approach aligns outcome assessment with priorities central to hematologic oncology care, where maintaining treatment schedules is often essential for disease control.

This study has multiple limitations, mostly due to its retrospective design. First, its design introduces the potential for residual confounding despite robust adjustment using IPTW. Treatment allocation was not randomized, and patients undergoing surgical evacuation presented with greater radiographic and neurologic severity, although balance across prespecified covariates was achieved after weighting. Nevertheless, the MMAE cohort were symptomatic with substantial cSDH, and the lower rate of rescue treatment is clinically important. Second, the limited sample size did not permit a subgroup analysis of specific malignancy subtypes or treatment regimens. Third, this study was conducted at a single tertiary cancer center, which may limit generalizability. Finally, while time to resumption of systemic therapy is a clinically meaningful surrogate endpoint, the impact of earlier treatment resumption on long-term oncologic outcomes, such as PFS or OS, was not assessed and warrants further investigation.

## 5. Conclusions

In patients with hematologic malignancies and cSDH, MMAE was associated with a lower need for rescue treatment and earlier resumption of systemic cancer therapy compared with surgical evacuation. These findings suggest that MMAE may represent a minimally invasive treatment strategy that aligns neurosurgical effectiveness with the imperative to preserve continuity of oncologic care in this high-risk population. Prospective multicenter studies are needed to define optimal patient selection and management of oncological treatment, and ultimately to evaluate potential survival benefits.

## Figures and Tables

**Table 1:** Demographic, clinical, and outcomes distribution across groups.

Variable	MMAE (*n* = 37)	Surgery (*n* = 30)	*p* Value
**Age, median (IQR)**	69.0 (59.0–76.0)	66.0 (57.0–71.8)	0.328 ^1^
**BMI, median (IQR)**	27.1 (23.2–31.6)	26.0 (23.2–30.5)	0.752 ^1^
**Sex**			0.215 ^2^
Female	17 (45.9%)	9 (30.0%)	
Male	20 (54.1%)	21 (70.0%)	
**KPS, median (IQR)**	60 (60–80)	75 (60–80)	0.273 ^1^
**Alcohol use**			0.749 ^3^
Never	33 (89.2%)	25 (83.3%)	
Current	3 (8.1%)	3 (10.0%)	
Former	1 (2.7%)	2 (6.7%)	
**Smoking status**			0.011 ^3^
Never	22 (59.5%)	21 (72.4%)	
Current	14 (37.8%)	3 (10.3%)	
Former	1 (2.7%)	5 (17.2%)	
**Renal Replace Therapy**	1 (2.7%)	2 (6.7%)	0.583 ^3^
**HIV positive**	0 (0.0%)	2 (6.7%)	0.197 ^3^
**Prior cerebro/cardiovascular disease**	13 (35.1%)	7 (23.3%)	0.421 ^2^
**Malignancy**			0.504 ^3^
Myeloid neoplasms	23 (60.5%)	15 (39.5%)	
B-cell lymphomas	6 (37.5%)	10 (62.5%)	
Lymphoid leukemias	4 (57.1%)	3 (42.9%)	
Plasma cell dyscrasias	3 (60.0%)	2 (40.0%)	
T/NK-cell lymphomas	1 (100.0%)	0 (0.0%)	
**Concurrent solid tumor**	7 (18.9%)	3 (10.0%)	0.493 ^3^
**Known prior head trauma**	5 (13.5%)	5 (16.7%)	0.743 ^3^
**Dizziness (pre-op)**	10 (27.0%)	14 (46.7%)	0.126 ^2^
**Fever**	2 (5.4%)	2 (6.7%)	1.000 ^3^
**Glasgow Coma Scale, median (IQR)**	15 (13–15)	15 (12–15)	0.205 ^3^
**Headache severity (pre-op)**			
Mild	24 (64.9%)	8 (26.7%)	
Moderate	0 (0.0%)	9 (30.0%)	
Severe	0 (0.0%)	7 (23.3%)	
**Motor deficit**	6 (16.2%)	7 (23.3%)	0.542 ^2^
**Seizures**	1 (2.7%)	0 (0.0%)	1.000 ^3^
**Sensory deficit**	1 (2.7%)	3 (10.0%)	0.318 ^3^
**Visual symptoms**	1 (2.7%)	4 (13.3%)	0.165 ^3^
**Currently on chemotherapy**	31 (83.8%)	24 (80.0%)	0.755 ^2^
**Hematocrit (%), median (IQR)**	26.3 (23.7–28.8)	27.9 (26.2–31.0)	0.033 ^1^
**INR, median (IQR)**	1.2 (1.2–1.3)	1.2 (1.1–1.4)	0.217 ^1^
**Platelets (×10^9^/L), median (IQR)**	77 (39–114)	84.5 (42.8–150.8)	0.442 ^1^
**PT, (s), median (IQR)**	15.1 (14.3–15.8)	15.1 (13.9–16.4)	0.989 ^1^
**aPTT, (s), median (IQR)**	31.2 (28.6–37.2)	30.1 (28.8–34.4)	0.621 ^1^
**Midline shift (mm), median (IQR)**	0.0 (0.0–5.4)	8.9 (7.0–12.2)	<0.001 ^1^
**cSDH thickness (mm), median (IQR)**	13.2 (10.4–16.1)	17.6 (14.5–21.4)	0.005 ^1^
**Preoperative Steroids**	6 (16.2%)	12 (40.0%)	0.051 ^2^
**Antiplatelet therapy**	1 (2.7%)	2 (6.7%)	0.583 ^3^
**Anticoagulation**	3 (8.1%)	2 (6.7%)	1.000 ^3^
**Preoperative coagulopathy**			<0.001 ^1^
Thrombocytopenia	5 (14%)	11 (37%)	
Prolonged coagulation times	2 (5.4%)	0 (0%)	
Mixed	28 (76%)	9 (30%)	
**Correction of coagulopathy**	1 (2.7%)	12 (40%)	<0.001 ^3^
**Preoperative Transfusion**	27 (75.0%)	21 (70.0%)	0.783 ^2^
**RBC Preop, median (Range)**	0.0 (0.0–5.0)	0.0 (0.0–4.0)	0.800
**Platelets units preop, median (Range)**	3.0 (0.0–7.0)	2.0 (0.0–12.0)	0.323 ^1^
**Postoperative units transfused, median (IQR)**	3.0 (0.8–5.0)	4.0 (0.0–10.0)	0.093 ^1^
**Postoperative RBC, median (Range)**	1.0 (0–2.0)	2.0 (0.0–4.0)	0.426 ^1^
**Postoperative Platelets, median (Range)**	1 (0–6)	2 (0–12)	0.27 ^1^
**Procedure duration (min), median (IQR)**	60.0 (45.0–60.0)	135.0 (96.2–177.5)	<0.001 ^1^
**Rescue treatment**	3 (8.1%)	10 (33.3%)	0.013 ^2^
**Time to rescue (days), median (IQR)**	5.0 (1.0–13.0)	11.0 (3.5–36.8)	0.012 ^1^
**Intraoperative complication**	0 (0%)	1 (3.3%)	0.448 ^3^
**New postoperative neurologic deficit**	3 (8.1%)	9 (30.0%)	0.027 ^3^
**Wound complication**	0 (0%)	3 (10.0%)	0.085 ^3^
**Any in-hospital complication**	4 (11%)	13 (43%)	0.004 ^3^
**Hospital length of stay (days), median (IQR)**	4 (2 to 9)	19 (9 to 29)	0.001 ^1^
**Mortality after index procedure**			
30-day	3 (8.1%)	0 (0%)	0.247 ^3^
90-day	13 (35.1%)	3 (10.0%)	0.022 ^3^
180-day	18 (48.6%)	7 (23.3%)	0.044 ^2^

MMAE = Middle Meningeal Artery Embolization; cSDH = Chronic Subdural Hematoma; KPS = Karnofsky Performance Status; INR = International Normalized Ratio; IQR = Interquartile Range; RBC = Red Blood Cell. ^1^ Wilcoxon rank-sum (Mann–Whitney U); ^2^ Pearson’s chi-square; ^3^ Fisher’s exact.

**Table 2:** Baseline balance (unweighted vs. weighted, by procedure).

Variable	Unweighted				Weighted			
	MMAE (*n* = 37)	Surgery (*n* = 30)	*p*	SMD	MMAE (*n* = 37)	Surgery (*n* = 28.2)	*p*	SMD
**Age ≥ 65 years**	23 (62.2)	15 (51.7)	0.548	0.212	60%	56%	0.72	0.098
**KPS > 70**	12 (32.4)	14 (48.3)	0.292	0.327	35%	39%	0.676	0.088
**Platelets**								
≤50 × 10^9^/L	13 (35.1)	9 (31.0)	0.917	0.103	35.1%	32.8%	0.964	0.091
50–100 × 10^9^/L	10 (27.0)	9 (31.0)	–	–	27.0%	31.9%	–	–
>100 × 10^9^/L	14 (37.8)	11 (37.9)	–	–	37.8%	35.3%	–	–
**INR > 1.5**	3 (8.1)	3 (10.3)	1	0.077	8%	9%	0.962	0.017
**Active cancer therapy**	31 (83.8)	23 (79.3)	0.88	0.116	84%	83%	0.976	0.009
**Antithrombotic therapy**	4 (10.8)	4 (13.8)	1	0.091	11%	11%	0.998	0.001
**Midline shift ≥ 5 mm**	10 (27.0)	25 (86.2)	<0.001	1.489	28%	33%	0.71	0.092
**Acute/mixed cSDH**	29 (78.4)	14 (48.3)	0.02	0.658	57%	52%	0.101	0.095

MMAE = middle meningeal artery embolization; cSDH = chronic subdural hematoma; SMD = standardized median difference.

**Table 3:** Weighted Cox proportional hazards—time to rescue treatment.

Variable		Univariate		Multivariate	
		HR (95% CI)	*p*-Value	HR (95% CI)	*p*-Value
**Procedure**	Surgery	ref		ref	
	MMAE	0.21 (0.06–0.78)	0.019	0.19 (0.05–0.81)	0.024
**Age**	<65 y	ref		ref	
	≥65 y	0.55 (0.18–1.63)	0.278	0.40 (0.11–1.47)	0.169
**Karnofsky performance score**	<70	ref		ref	
	>70	1.18 (0.38–3.61)	0.774	2.72 (0.77–9.60)	0.121
**Midline shift**	<5 mm	ref		ref	
	≥5 mm	2.32 (0.71–7.54)	0.161	0.95 (0.23–3.88)	0.942
**Preoperative INR**	<1.5	ref		ref	
	>1.5	1.82 (0.40–8.22)	0.438	1.23 (0.18–8.26)	0.828
**Preoperative PTT**	<35 s	ref		ref	
	>35 s	1.99 (0.65–6.09)	0.227	5.38 (1.07–12.19)	0.041
**Preoperative platelets**	>100 × 10^9^/L	ref		ref	
	50–100 × 10^9^/L	0.52 (0.16–1.72)	0.287	0.49 (0.12–1.91)	0.302
	≤50 × 10^9^/L	0.24 (0.05–1.21)	0.085	0.16 (0.03–0.87)	0.034

## Data Availability

Data is available per explicit and direct inquiry to the corresponding author.

## Supplementary Materials

The following supporting information can be downloaded at https://www.mdpi.com/article/10.3390/cancers18172824/s1, Figure S1: (A) Propensity score overlap. (B) Covariate balance; Table S1: Individualized data, diagnosis, platelets and systemic therapy characteristics.

## Abbreviations

The following abbreviations are used in this manuscript:

MMAE	Middle Meningeal Artery Embolization
cSDH	Chronic Subdural Hematoma
MMA	Middle Meningeal Artery
IPTW	Inverse Probability of Treatment Weighting
HR	Hazard Ratio
CI	Confidence Interval
aPTT	Activated Partial Thromboplastin Time
PT	Prothrombine Time
INR	International Normalized Ratio
KPS	Karnofsky Performance Status
IQR	Interquartile Range
PFS	Progression-Free Survival
OS	Overall Survival
CT	Computed Tomography
MRI	Magnetic Resonance Imaging
GCS	Glasgow Coma Scale
BMI	Body Mass Index
HIV	Human Immunodeficiency Virus
