# Supplementary material for: Preserving Continuity of Cancer Therapy in Patients with Hematologic Malignancies and Chronic Subdural Hematomas: Comparative Outcomes of Middle Meningeal Artery Embolization and Surgery
Source: Cancers (Basel). 2026 Sep 1;18(17):2824. doi: 10.3390/cancers18172824 (PMC13564524; doi:10.3390/cancers18172824)
Supplement: Supplementary file 1 [file cancers-18-02824-s001.zip › cancers-4419734-Figure S1.pdf]

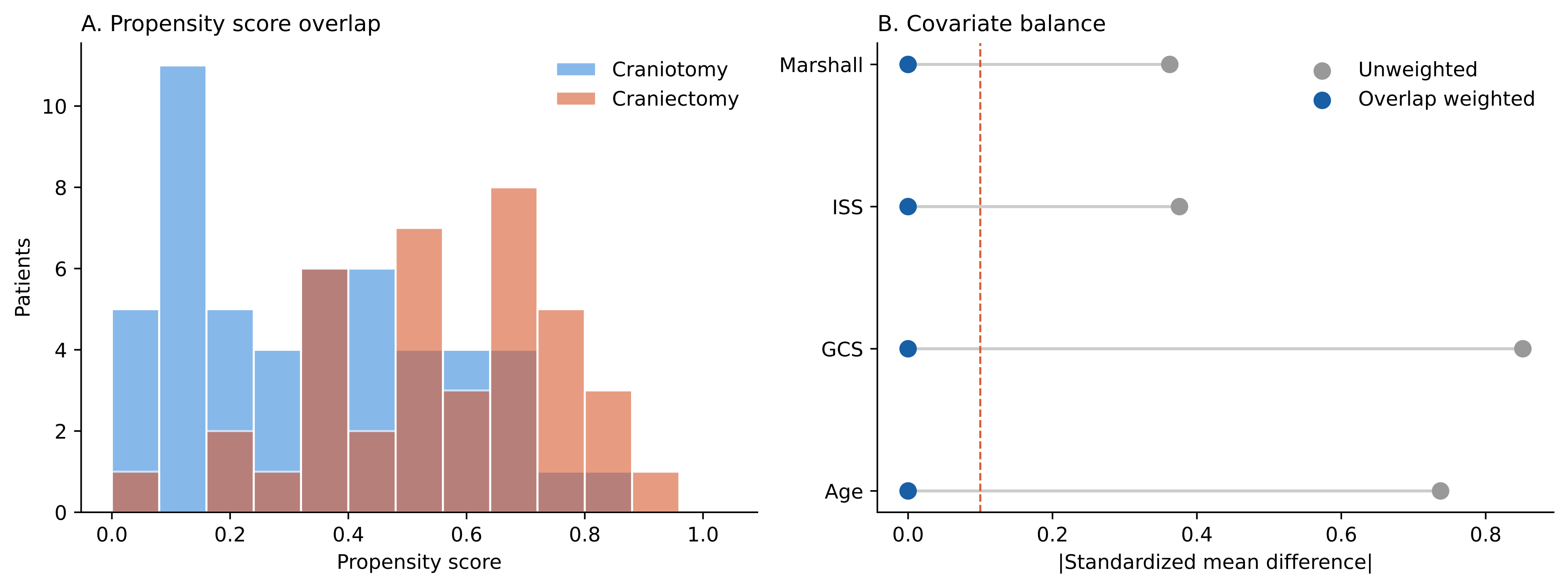

A. Propensity score overlap
Craniotomy
Craniectomy
Patients
Propensity score
0
2
4
6
8
10
0.0
0.2
0.4
0.6
0.8
1.0
B. Covariate balance
Unweighted
Overlap weighted
Marshall
ISS
GCS
Age
0.0
0.2
0.4
0.6
0.8
|Standardized mean difference|
