# Supplementary material for: Preserving Continuity of Cancer Therapy in Patients with Hematologic Malignancies and Chronic Subdural Hematomas: Comparative Outcomes of Middle Meningeal Artery Embolization and Surgery
Source: Cancers (Basel). 2026 Sep 1;18(17):2824. doi: 10.3390/cancers18172824 (PMC13564524; doi:10.3390/cancers18172824)
Supplement: Supplementary file 1 [file cancers-18-02824-s001.zip › cancers-4419734-Table S1.pdf]

Supplementary Table S1. Individualized data, diagnosis, platelets and systemic therapy characteristics.

Patient	Procedure	Specific diagnosis	Preop platelets (×10⁹/L)	Preprocedural platelet units	On systemic therapy	Therapy was held for cSDH	Time to restart therapy (d)	Rescue treatment	Systemic therapy regimen
**1**	MMAE	Myelodysplastic syndrome	70	6	No	No		No	None
**2**	MMAE	Acute myeloid leukemia	45	3	No	No		No	None
**3**	MMAE	Myelodysplastic syndrome	77	4	Yes	Yes	28	No	Venetoclax and ASTX727
**4**	MMAE	Acute lymphoblastic leukemia	108	0	Yes	Yes	8	No	Blinatumomab
**5**	MMAE	Multiple myeloma	93	3	Yes	Yes	13	Yes	Daratumumab, Bortezomib, Cyclophophamide and Dexamethasone
**6**	Surgery	Mantle cell lymphoma	48	3	Yes	No		No	Bendamustine + Rituxan
**7**	MMAE	T-cell lymphocytic leukemia	116	1	Yes	Yes	8	No	Decitabine
**8**	Surgery	Acute myeloid leukemia	92	1	Yes	Yes	34	Yes	Venetoclax, Gilteritinib, Azacitidine
**9**	Surgery	Acute myeloid leukemia	65	1	Yes	Yes	104	Yes	Inotuzumab + Blinatumomab
**10**	MMAE	Acute myeloid leukemia	127	0	Yes	Yes	17	No	Cladribine and Low-Dose Cytarabine Alternating with Decitabine
**11**	MMAE	Blastic plasmacytoid dendritic cell neoplasm	114	4	Yes	Yes	9	No	Cytarabin + Metothrexate intrathecal
**12**	MMAE	Large B-cell lymphoma	270	0	Yes	No		No	rituximab, ifosfamide, carboplatin, and etoposide
**13**	MMAE	Acute lymphocytic leukemia	93	0	Yes	Yes	14	No	Mini CVD + Inotuzumab + Vincristine
**14**	Surgery	Large B-cell lymphoma	151	0	No	No		No	None
**15**	Surgery	Acute lymphocytic leukemia	301	2	No	No		No	None
**16**	Surgery	Acute Myeloblastic leukemia	84	2	Yes	Yes	23	Yes	Idarubicin
**17**	Surgery	Acute lymphocytic leukemia	41	2	Yes	Yes	9	No	Avapritinib
**18**	Surgery	Acute myeloid leukemia	201	0	Yes	Yes	37	No	ludarabine and Melphalan 140 with Post Transplant Cyclophosphamide
**19**	Surgery	Large B-cell lymphoma	150	6	No	No		No	None
**20**	Surgery	Acute myeloid leukemia	98	6	Yes	Yes	18	Yes	APR-246, Venetoclax, Azacitidine
**21**	Surgery	Acute myeloid leukemia	3	6	Yes	Yes	13	No	Cytarabin
**22**	Surgery	Acute myeloid leukemia	124	6	Yes	No		Yes	Azacitidine, Ipilimumab, and Nivolumab
**23**	Surgery	Acute myeloid leukemia	81	4	Yes	Yes	27	No	Azacitidine, Ipilimumab, and Nivolumab
**24**	Surgery	Follicular lymphoma	134	0	No	No		No	None

**25**	Surgery	Chronic myeloid leukemia	30	6	Yes	Yes	22	No	FIA + Bosutinib
**26**	Surgery	Mantle cell lymphoma	131	0	No	No		No	None
**27**	Surgery	Acute myeloid leukemia	574	0	Yes	Yes	56	Yes	Dasatinib
**28**	Surgery	Follicular lymphoma	294	0	Yes	Yes	62	Yes	Methotrexate + R-IE
**29**	Surgery	Mantle cell lymphoma	261	0	Yes	Yes	18	Yes	R-EPOCH + Velcade
**30**	Surgery	Acute myeloid leukemia	83	2	Yes	Yes	26	Yes	Venetoclax, Gilteritinib, Azacitidine
**31**	Surgery	Acute myeloid leukemia	16	6	Yes	Yes	27	No	Decitabine
**32**	Surgery	Multiple myeloma	85	12	Yes	Yes	50	No	Sotatercept
**33**	Surgery	Myelodysplastic syndrome	10	12	Yes	Yes	47	No	Guadecitabine
**34**	Surgery	Acute myeloid leukemia	16	6	Yes	Yes	17	No	PKC412 and 5-azacitidine
**35**	Surgery	Multiple myeloma	55	6	Yes	Yes	44	No	Lenalidomide and Azacitidine
**36**	Surgery	Follicular lymphoma	249	0	Yes	Yes	19	No	R-EPOCH
**37**	Surgery	Follicular lymphoma	3	12	Yes	Yes	389	No	Bendamustine/Rituxan
**38**	Surgery	Acute myeloid leukemia	273	0	Yes	Yes	8	No	HyperCVAD + Dasatinib
**39**	Surgery	Follicular lymphoma	60	6	No	No		No	None
**40**	MMAE	Chronic myeloid leukemia	12	0	Yes	Yes	32	No	Imatinib
**41**	MMAE	T-cell lymphocytic leukemia	31	6	Yes	Yes	34	Yes	miniCVD + Venetoclax
**42**	MMAE	Chronic lymphocytic leukemia	228	0	No	No		No	None
**43**	MMAE	Acute myeloid leukemia	68	6	Yes	Yes	10	No	Cladribine, low-dose cytarabine, decitabine
**44**	MMAE	Multiple myeloma	59	3	No	No		No	None
**45**	MMAE	T-cell lymphoma	115	0	Yes	No		No	Intrathecal + Ara-C
**46**	MMAE	Myelodysplastic syndrome	5	3	Yes	Yes	15	No	Cladribine, low-dose cytarabine, decitabine
**47**	MMAE	Myelodysplastic syndrome	109	2	Yes	Yes	9	No	Azacitidine + Venetoclax
**48**	MMAE	Acute myeloid leukemia	101	0	Yes	Yes	9	No	Busulfan
**49**	MMAE	Non-Hodgkin lymphoma	214	0	No	No		No	None
**50**	MMAE	Chronic myeloid leukemia	40	4	Yes	Yes	6	No	CAT + Ponatinib
**51**	MMAE	Acute myeloid leukemia	17.3	7	Yes	Yes	14	No	Cladribine, low-dose cytarabine, decitabine
**52**	MMAE	Mantle cell lymphoma	18	4	Yes	Yes	2	No	Rituximab + Zanubrutinib
**53**	MMAE	Multiple myeloma	138	0	Yes	Yes	26	No	Isatuximab, Bortezomib, and Dexamethasone
**54**	MMAE	Acute myeloid leukemia	31	3	Yes	Yes	6	No	APR-246, Venetoclax, Azacitidine
**55**	MMAE	Large B-cell lymphoma	190	0	Yes	Yes	6	Yes	DA-REPOCH + IT

**56**	MMAE	Large B-cell lymphoma	19	3	Yes	Yes	55	No	Cytarabin
**57**	MMAE	B-cell lymphoma	236	0	Yes	No		No	RCHOP + IT
**58**	MMAE	Acute myeloid leukemia	108	2	Yes	Yes	3	No	Fludarabine, Idarubicin, Cytarabine, Venetoclax, Dexamethasone, Vincristine, Blinatomomab and Pegaspargase
**59**	MMAE	Acute myeloid leukemia	78	3	Yes	Yes	15	No	Cladribine and Cytarabine
**60**	MMAE	Acute myeloid leukemia	60	5	Yes	Yes	5	No	Venetoclax and S64315 trial
**61**	MMAE	Acute myeloid leukemia	62	2	Yes	Yes	33	No	Azacitidine + Venetoclax
**62**	MMAE	Myelodysplastic syndrome	98	2	Yes	No		No	Busulfan, Fludarabine, Cladribine
**63**	MMAE	Myelodysplastic syndrome	39	0	Yes	Yes	89	No	Azacitidina/Cedazirudine
**64**	Surgery	Myelodysplastic syndrome	24	2	Yes	Yes	25	Yes	Decitabine, Cedazuridine
**65**	MMAE	Acute myeloid leukemia	45	5	Yes	No		No	VIP943
**66**	MMAE	Chronic myeloid leukemia	38	6	No	No		No	None
**67**	MMAE	Acute myeloid leukemia	18	4	Yes	Yes	22	No	Cytarabine
